# Celluloid Manufacturing Company

**Published:** 1875-07

**Authors:** 


*Celluloid Manufacturing Company* versus *Goodyear Dental Vulcanite Company.*—The following correspondence will prove interesting to our readers:

"Dear Sir:—We have received numerous letters from members of the dental profession, informing us that Josiah Bacon, or his agents, seek to interfere with the use of Celluloid as a base for artificial teeth, by asserting that such use is an infringement of the Cummings patent, and by threats of prosecution against all dentists who use Celluloid and refrain from taking out a license from the Goodyear Dental Vulcanite Company.

We caution the profession against being intimidated or deceived by such statements. The use of Celluloid is not an infringement of the Cummings patent; and as for threatened prosecution, we guarantee protection to all dentists in the use of Celluloid against any legal assault that may be made against them on that account.

The following opinion by our counsel, Henry Baldwin, Jr., Esq., warrants us in publishing this guarantee, and we have no doubt will satisfy all who read it that the statements above referred to, made in the interest of rubber licenses, are false and fraudulent."

Yours truly,

CELLULOID MFG. COMPANY, NEWARK, N. J.

---

217 S. SIXTH ST., PHILADELPHIA, JUNE 1ST., 1875.

*The Celluloid Manufacturing Co.:*

*Gentlemen:*—You submit to me the following letter, viz:

GOODYEAR DENTAL VULCANITE CO., Josiah Bacon, Treasurer.

*No.* 5 *Temple Place,* 38 *Park Row, New York.*

*Boston, Mass.*

BOSTON. FEBRUARY, 15TH, 1875.

*Dr. S. H. Wirts, Steel Mills, Ills.*

"DEAR SIR:—Yours of the 12th has my attention. Permit me to inform you that the 'Smith case' to which you so aptly allude, has been decided in my favor, and you will find the Supreme Court at Washington will but *confirm* that decision.

Permit me also to inform you that the use of Celluloid is an infringement of our Patent, it being the 'equivalent' of Rubber as stated in our Patent, copy of which I enclose."

Yours truly,

(Signed) JOSIAH BACON, Treas.

"And you ask my opinion as to the truth of the assertion made in the last paragraph 'that the use of Celluloid is an infringement' of the Patent indicated.

I understand the Patent referred to as being the reissue No. 1,904, dated March 21st, 1865, and known as the Cummings Patent, that being the only Patent involved in the Smith case.

In that case the testimony of the Goodyear Dental Vulcanite Company's expert, Mr. Henry B. Renwick, was that in order to get a Cummings set of teeth, you must first have a vulcanizable compound of gum and sulphur, and finally vulcanite. The arguments of their counsel accepted this definition of what is embraced in the Reissue No. 1,904, and the opinion of the Court adopts this construction of the Cummings Patent.

The claim of the Patent is 'The plate of hard rubber or vulcanite, or its equivalent, for holding artificial teeth, or teeth and gums, substantially described.' The specification does not mention any material to be used for forming the plate except soft rubber, or gum, compounded with rubber, sulphur, &c., 'in the manner prescribed in the Patent of Nelson Goodyear, dated May 6th, 1851, for making hard rubber, and is to be subjected to sufficient heat to harden it, substantially as directed in that Patent.' 'After the plate has been heated sufficiently to harden it, or convert it into hard rubber, or vulcanite so-called, the mould is removed and the plate is polished ready for use.'

Any vulcanized plate made from any vulcanizable compound would be comprised in the claim under the words 'hard rubber or vulcanite or its equivalent;' but no plate not vulcanized or made of vulcanizable compound, would come within the claim of the Cummings Patent.

Celluloid contains neither rubber nor any other vulcanizable gum, nor sulphur nor any other vulcanizing agent. Plates made of Celluloid are neither vulcanized nor vulcanizable, and it is not true that the use of Celluloid is an infringement of the Cummings Patent, Reissue No. 1,904, dated March 21st, 1865.

Referring to certain other letters also submitted to me, in which I find it stated that agents of the Goodyear Dental Vulcanite Company are demanding money and claiming license fees under this Cummings Patent Reissue No. 1,904, for the past use of Celluloid, alleging that such use was an infringement of that Patent, I would suggest that each Dentist should protect himself against such unlawful exactions or attempts, by having any person enforcing such demand or claim arrested and dealt with by the proper local authorities, for obtaining money under false pretences."

Respectfully,

HENRY BALDWIN, JR.. *Counsellor at Law.*

